# An in-vitro study on effects of laser activation on dye penetration in human root dentin

**DOI:** 10.2340/biid.v11.40311

**Published:** 2024-04-05

**Authors:** Clara Isabel Anton y Otero, Laurine Marger, Enrico di Bella, Albert Feilzer, Ivo Krejci, Marwa Abdelaziz

**Affiliations:** a Division of Cariology and Endodontology, CUMD – University Clinics of Dental Medicine, Faculty of Medicine, University of Geneva, Geneva, Switzerland; b Department of Dental Material Sciences, Academic Center for Dentistry Amsterdam (ACTA), Amsterdam, The Netherlands

**Keywords:** Laser-activated irrigation, disinfection, cleaning, irrigation, laser

## Abstract

**Objective:**

To evaluate the penetration of a dye in root dentin after activation with different laser wavelengths.

**Materials:**

Palatal roots of 38 human molars were enlarged and disinfected. Irrigation activation was performed with an Er:YAG laser: @50 mJ, 15 Hz (Er:YAG); a 9.3 µm CO_2_ laser: @40% power (CO_2_); diode lasers 455 nm/970 nm: @0.8 W, 15 Hz (D455, D970) and 808/980 nm: @1 W (D808, D980) and compared to positive control: etching with 35% H_3_PO_4_ (POS); negative control: water (NEG) and conventional needle irrigation: NaOCl and ethylenediaminetetraacetic acid (EDTA) (CONV). Methylene blue solution was introduced in the canal and laser-activated or left untouched for 100 s before the roots were dried and cut into horizontal slices. Dye penetration was automatically calculated by color recognition of two samples per root third (*n* = 8 per group in each coronal, middle and apical root thirds). The presence and absence of a smear layer was checked in two additional samples of the negative and positive control under scanning electron microscopy (SEM).

**Results:**

Full-depth infiltration was not achieved in any group. Dye penetration in CONV was significantly less than in Er:YAG, CO_2,_ POS, D455, D970, D808 and similar to NEG and D980 when results of different root thirds were pooled.

**Conclusion:**

Laser activation using certain parameters enhanced dye penetration compared to conventional needle irrigation with NaOCl and EDTA (CONV).

## Introduction

Endodontic infections are driven by bacteria that colonize the necrotic pulp tissues and the surfaces of dentinal root walls as well as penetrate into the dentin through the tubules up to a depth of 2,000 µm [1].

Clinical protocols for endodontic treatments aim to eliminate a maximum of bacteria from the root canal surface including the dentinal tubules. The protocols are based on the mechanical removal of pulpal tissues and the infected interior dentin layers with files and reamers and on disinfection by rinsing with irrigants [2, 3].

The mechanical preparation of the main root canals leads to the formation of debris and smear layer that covers the dentin surface with a thickness of 1–2 μm, but can also be packed inside the dentinal tubules up to a depth of 40 μm [4, 5]. Literature reports that the smear layer, containing microorganisms and their by-products, is prone to hinder disinfecting irrigants to access deep dentin layers [6] and additionally adversely affects the adaptation and adhesion of root canal sealers [7].

To address this issue, clinical protocols include not only the mechanical preparation, but also cleaning of the root surfaces from the smear layer and debris with chelating agents such as ethylenediaminetetraacetic acid (EDTA), and reduction of bacteria within the tissues with disinfecting agents such as for example sodium hypochlorite (NaOCl).

Literature has shown that the degree of penetration of irrigants to scavenge bacteria residing deeply inside infected dentinal tubules is more important than the instrumentation of the root canal systems itself [8]. However, with traditional syringe irrigation the apical parts of root canal systems and deep dentin layers are often not reached, leading to persistent periradicular disease [9]. Conventional needle irrigation allows only NaOCl infiltration up to a depth of 250 µm into the tubules of root dentin [10] This might be due to chemical properties of irrigants such as the high surface tension as well as the limited streaming effects of the irrigation method [11, 12].

To improve the penetration of irrigants, several devices and methods have been developed, such as ultrasonics or lasers [13]. Laser activated irrigation (LAI) employs cavitation to generate streaming of the irrigant, resulting in mechanical cleaning of the root wall surfaces. In-vitro research with erbium lasers has shown promising results of LAI with chelating agents as for instance EDTA for the removal of the superficial smear layer and opening of dentinal tubule as well as to bacteria reduction on the superficial root dentin walls [14, 15]. However, the pure mechanical effect of LAI with these lasers seems to be concentrated on promoting the irrigant to travel in the apical region and less to detach smear layer from the root canal walls [16]. The impact of the pure effects of laser activation on deep root dentin layers is not clear until recently.

Diode lasers are more and more accepted and used in periodontics for pocket disinfection. These systems are significantly smaller and cost attractive than established erbium lasers. In endodontics, diode lasers were basically applied for laser light irradiation of the root canal walls that lead, depending on the wavelength and absorption capacities of the tissues, to thermal heating of the bacteria and their environment [17]. Literature reports on significant bacteria reduction up to 1 mm dentin depth and also on important thermal damage to root dentin and periapical tissues dependent on the wavelength and the power applied [18]. These findings explain why this method has not been established in clinical protocols.

Diode laser light is not absorbed in water and is therefore not suitable for the activation of transparent endodontic irrigants. However, when laser tips are coated black with for example, carbon powder, the laser energy is absorbed in this black layer. The coated laser tip heats up and increases the temperature of the surrounding liquid until it starts to boil and forms vapor bubbles. The implosion of these bubbles leads to secondary cavitation and introduces movements of the liquid [19].

Recently, a new 9,300 nm CO_2_ laser system became popular in its application for dental hard tissue and surgical treatments. Research showed promising results for activation of endodontic irrigants [19].

In artificial root models it has been shown that LAI with diode and 9,300 nm CO_2_ lasers with specific power parameters enhance the liquid penetration in accessory side canals more than traditional needle irrigation and to a degree comparable to Er:YAG laser activation [20].

However, it is not clear whether the mechanical effects of LAI in liquids seen in relatively large side canals also promote liquid penetration via narrow tubules into deep dentin layers.

This study aimed to investigate the capacity of LAI with a new 9,300 nm CO_2_ and low power diode lasers to enhance dye penetration into deep dentin layers of root canal systems covered with a heavy smear layer and to compare pure laser effects of diode and CO_2_ lasers to Er:YAG laser activation and to traditional needle irrigation with chelating agents. The first null hypothesis stated that LAI with the tested lasers would be at least as effective as or superior to traditional needle irrigation with chelating agents in dentinal tubule penetration depth. The second null hypothesis stated that CO_2_ and diode laser activation would be similar to Er:YAG laser activation.

## Materials and methods

### Root canal preparation

The sample size calculations were based on results of unpublished preliminary tests that indicated a surface ratio of penetration in horizontal root slices with methylene blue of 1.7 (SD 1.0) in the positive control and the 0.4 (SD 0.3) in conventional syringe irrigation groups. With a power of 80% and a two-sided alpha error of 5% we calculated a required sample size of *n* = 8 specimens per root third.

To prepare the samples, 38 caries-free human maxillary third molars with completed apex and straight palatal roots were selected from a pool of anonymized extracted teeth from the surgical department of the University clinics of Geneva (HUG-Hopitaux Universitaires Genève, chirurgie maxillo-faciale et buccale). The local ethical committee considers pooled biobanks as irreversibly anonymized and waives the necessity for ethical approval. Teeth were stored after extraction at 6°C in a water-based solution of 0.02 g/mL thymol (Sigma-Aldrich, Steinheim, Germany)

Teeth with apical diameters of larger than size 15 taper C-pilot file (K Files, MICRO-MEGA, Besançon Cedex, France) and complex anatomical structures such as palatal side canals, resorptions, or root curvatures were excluded. Teeth were cleaned from adhering soft-tissue remnants with an ultrasonic scaler (EMS Piezo Master 400, EMS SA Nyon, Switzerland).

Working length was determined as visual patency length minus 1 mm. To standardize the root length, the teeth were sectioned 12 mm from the apex with a diamond disc (127 mm diameter, ×0.4 mm, Cut-off Wheel MOD 13, Struers, Ballerup, Denmark) and an access cavity to the pulpal tissues was finished with a diamond bur (25 µm, Intensiv, Montagnola, Switzerland).

Except for roots of the positive control group (POS), root apices were sealed with a flowable composite (Tetric EvoFlow, Ivoclar Vivadent, Schaan, Liechtenstein) without adhesive procedures under a stereomicroscope of magnification ×16 (Leica, Wild, Heerbrugg, Switzerland) in order to create a closed-ended channel for later applied activation and hermeticity controlled. To avoid dye penetration from the external root surface, the roots were covered with two layers of nail varnish (SUPER STAY 7 days, Maybelline, New York City, NY, USA).

### Shaping and cleaning

Pulp tissue remnants were then removed with a size 15 taper C-pilot file (K Files, MICRO-MEGA, Besançon Cedex, France) and root canals were prepared using a crown down technique with a rotary file system (ProTaper Gold, Maillefer Dentsply, Ballaigues Switzerland) and an endodontic motor (x smart plus, Maillefer Dentsply, Ballaigues, Switzerland) up to a diameter of F4 (0.40/0.6 v).

**Figure 1:**
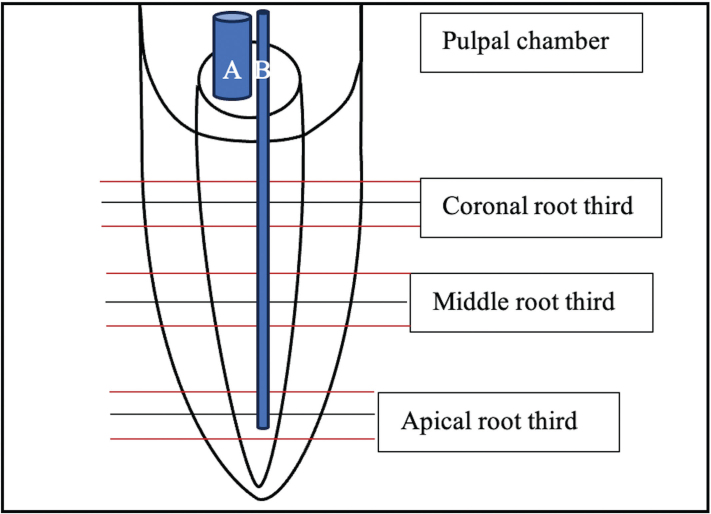
Overview over a root sample and positions of horizontal cuts and laser tips: A: Er:YAG /CO_2_ laser; B: diode lasers.

Subsequently, the root canals were subjected to a specific rinsing protocol (Table 1). To investigate the pure effects of laser activation, ultrapure water was applied with an open-ended endodontic needle (ENDO 30G, Transcodent, Kiel, Germany) as an irrigation liquid in the laser groups for both the mechanical preparation steps and for laser activation. For laser activation, the liquid was injected into the root main canal with the same needle and also filled the pulpal chamber. Er:YAG (Light Touch, Light Instruments Ltd, Yokneam, Israel) and CO_2_ laser (Solea, Convergent dental, Waltham, MA, USA) tips were activated at the root canal entrance while the tips of diode laser SIROLASE (Sirona dental systems GmbH, Bensheim, Germany) and diode laser WISER (Lambda SpA, Brendola, Italy) were inserted up to the working length minus 2 mm and moved in coronal to apical direction in continuous helical movements at 1 mm/s (see Figure 1). Diode laser tips were coated black prior to use with carbon particles (Carbon black, acetylene 100%, compressed, 99.9+%, Alfa Aesar, Kandel, Germany) that were glued with a transparent glue-spray (Toolcraft, Conrad Electronic AG, Wollerau, Switzerland) [19]. During activation, the liquid level in the access cavity was constantly controlled and replenished when needed.

**Table 1:** Description of the experimental groups with applied laser parameters and final irrigation procedure.

Group	Description	Irrigation procedure	Final methylene blue application
POS	Positive control	Rinsing with ultrapure water during instrumentation (10 mL), drying the canal with paper points (Large, WaveOne Gold, Dentsply Sirona, Baden, Switzerland), etching the canal walls with 35% H_3_PO_4_ (Ultra-Etch, Ultradent, South Jordan, UT, USA) for 60 s under constant movement with a paper-point and verifying that excess etching gel extruded the apex. Rinsing with ultra-pure water (5 mL)	100 s
NEG	Negative control (smear layer)	2 mL ultra-pure water between each file and a final rinse with 5 mL ultra-pure water	100 s
CONV	Conventional syringe irrigation	2 mL NaOCl (3%, Hänseler Swiss Pharma, Herisau, Switzerland) between each file followed by 5 mL EDTA (17%, Pharma24 SA, Geneva, Switzerland) and 5 mL ultra-pure water	100 s
Er:YAG	ER:YAG laser ((AS7066(X), 1.3×14 mm; 50 mJ, 15 Hz	2 mL ultra-pure water between each file	5× 20 s of laser activation
CO_2_	9.3 µm CO_2_ laser (Ultra-guide hand piece with endo-tip Ø 1.25 mm; 40%)	2 mL ultra-pure water between each file	5× 20 s of laser activation
D455	SIROLASE 455 nm (black coated tip Ø 0.4 mm; 0.8 W, 40% duty cycle, 15 Hz)	2 mL ultra-pure water between each file	5× 20 s of laser activation
D970	SIROLASE 970 nm (black coated tip Ø 0.4 mm; 0.8 W, 40% duty cycle, 15 Hz)	2 mL ultra-pure water between each file	5× 20 s of laser activation
D808	WISER 808 nm (black coated tip Ø 0.2 mm; 1 W, 26,666 µs on, 40,000 off)	2 mL ultra-pure water between each file	5× 20 s of laser activation
D980	WISER 980 nm (black coated tip Ø 0.2 mm; 1 W, 26,666 µs on, 40,000 off)	2 mL ultra-pure water between each file	5× 20 s of laser activation

EDTA: ethylenediaminetetraacetic acid; H3PO4: Phosphoric acid; NaOCl: Sodium hypochlorite

Irrigation in the positive and negative control was also performed with ultra-pure water applied with an 30G open-ended endodontic needle up to working length minus 1 mm. Canal walls of the positive control group were etched with phosphoric acid after mechanical preparation to open dentinal tubules to a maximum. The conventional group was rinsed with NaOCl during instrumentation and received a final rinse with EDTA and NaCl [19].

After the group-specific activation and rinsing, methylene blue solution (0.05 wt. % in water, Sigma-Aldrich) was applied for 100 s in each group.

In order to validate the preparation steps of the samples, one additional sample of the positive and the negative control, respectively, served for electron microscopy examination and confirmation of either a clean root canal surface or a heavy smear layer. With the aim to split the roots, a vertical groove was prepared at an external surface of the roots with a diamond disk (Diamond Cut-off Wheel M1D13, 127 mm dia. × 0.4 mm, Struers, Ballerup, Denmark) under constant water cooling. The roots were then split in a longitudinal direction along the root canal with a hammer and chisel into two halves and fixed in a solution of 0.1 M cacocylate (Sodium cacodylate trihydrate ≥ 98%, Sigma-Aldrich Chemie, Steinheim, Germany) and 2.5% glutaraldehyde (glutaraldehyde solution 50%, Sigma-Aldrich Chemie, Steinheim, Germany). Samples were dried in increasing concentrations of ethanolic solutions (Ethanol, EMSURE, Merck KGaA, Darmstadt, Germany) and fixed on custom made specimen holders with double-sided tape and then further dried in a vacuum desiccator (Kartell S.p.A., Noviglio, Italy) for 3 days. The fixed samples were then gold sputtered (a layer of 50 nm thickness with 24 karat gold, HHV Ltd, Crawley, UK) and subjected to SEM (Zeiss Gemini – Sigma 300 VP, Karl Zeiss Microscopy, Cambridge, UK) for examining the surface pattern of the internal root main canal surface in the central region over the entire root length at a magnification of 1,000×.

### Preparation for Keyence microscopy analysis

After 100 s, the methylene blue dye was removed from the root main canals with paper points (Large, WaveOne Gold, Dentsply Sirona, Baden, Switzerland), and teeth were air-dried inside and outside and samples where then stored for 3 days in a vacuum desiccator (Kartell S.p.A., Noviglio, Italy) until further use.

The apical 1 mm was removed and consistent sections of the root were made at the apical middle and coronal root thirds, each in triplicate (i.e. three 300 µm slices with a 600 µm distance between the root thirds) with a diamond disk (Diamond Cut-off Wheel M1D13, 127 mm dia. × 0.4 mm, Struers, Ballerup, Denmark lubricated with ISOCUT fluid, Buehler, Lake Bluff, IL, USA) (Figure 1).

Two non-consecutive specimens, each of 300 µm thickness, were obtained from each root third, dried and gently cleaned by rubbing between paper tissues. These specimens were then subjected to quantitative analysis using a digital microscope (KEYENCE VHX S5505E, Osaka, Japan).

**Figure 2:**
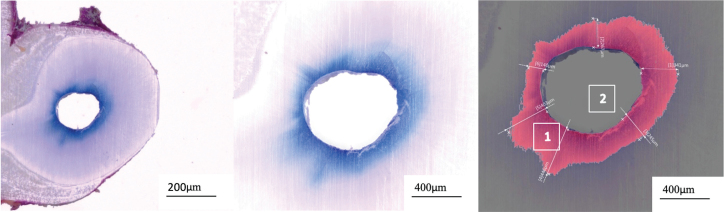
A: Overview (×50) of a horizontal slice from the middle root third of group D980; B: close-up (×200) of the dye penetrated area around the root main canal; C: automatic color recognition of the methylene blue dye (1) that was then divided by the root canal lumen surface for standardization (2) and measurement of the maximal dye penetration depth.

**Figure 3:**
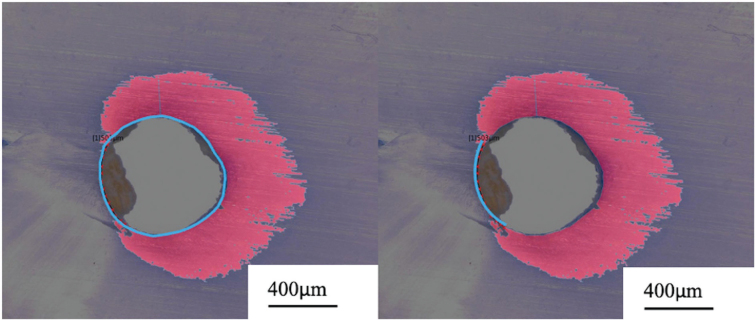
Photograph of a horizontal root cut out of the middle root third of group D970 (×200) and measuring of the root canal lumen slope (A) and the length of the slope without any dye penetration (B).

A total of 216 samples (24 samples per group) were analyzed. Slices were photographed at a magnification of 200×. The surface area of the root canal lumen and the surface area of dye penetration into the tubules were automatically calculated using a color recognition system (in µm^2^) of the microscope (Figure 2). To standardize the measurements, the surface area of dye penetration (Figure 2, right image, area number 1) was divided by the surface area of the canal lumen (area number 2). The maximal and minimal depth of dye penetration for each slice was also measured using the same measuring tool (Figure 2, right image). In addition, the length of the root canal lumen without any dye penetration (Figure 3, right image) was measured using a measuring tool and divided by the total length of the lumen (Figure 3, left image).

### Statistics

Statistical analyses were performed by means of two separate ANOVA for a full factorial design using penetration area and lack of penetration as dependent variables and Method and Region as main effects. The normal distribution assumption required to run ANOVA was checked by the Kolmogorov–Smirnov Normality test and Levene’s test for homogeneity of variances. Rankings among methods, regions and methods × regions combinations were assessed by means of Fisher’s LSD post hoc test. Statistical significance levels were set at 0.05. All the analyses were made in Minitab 19 by Minitab LLC.

## Results

The results demonstrated that dye penetration into the dentin varied depending on the experimental setup. Differences were observed among groups in terms of the percentage of penetration of the canal lumen scope, the area of penetration and the maximal penetration depth.

### Measurements of dye penetration along the canal lumen scope (homogeneity)

The ANOVA test detected a statistically significant effect on dye penetration along the canal lumen scope of the root regions (*p* < 0.001) but not of the activation method when all values of the different root thirds were grouped (*p* = 0.098).

**Figure 4:**
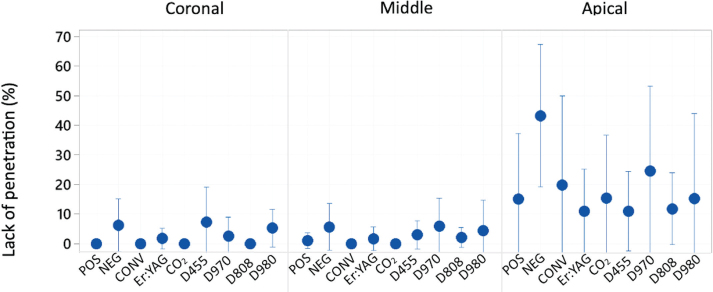
Interval plot representing a confidence interval of 95% for the mean of not penetrated percentage of the canal lumen scope in the coronal, middle and apical root part.

Means and 95% confidence intervals of dye penetration along the main root canal for each root third are presented in Figure 4. The apical third showed significant differences in dye penetration homogeneity compared to the middle and coronal root thirds, which were consistent across all groups.

The post-hoc analyses showed that the negative control group (NEG), where the root walls were covered with a heavy smear layer, exhibited a significantly higher percentage of unpenetrated parts of the canal lumen scope over the entire root length compared to the other experimental groups. The Sirolase 970 nm group (D970) had intermediate values that overlapped with both the negative control and the other groups. Conventional needle irrigation and laser activation with different wavelengths were similar (Figures 4 and 6).

**Figure 5:**
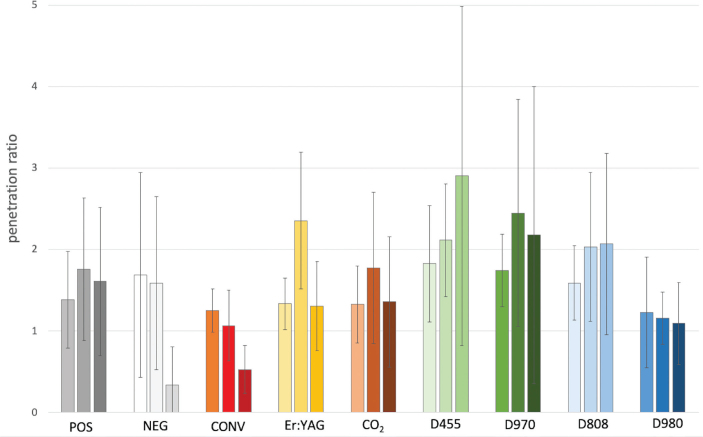
Mean dye propagation in the ratio of dentin surface area penetrated with the dye divided by the surface area of the canal lumen. Bars 1 to 3 in each group represent means in different root thirds; first bar: coronal root third; second bar: middle root third, third bar: apical root third.

**Figure 6:**
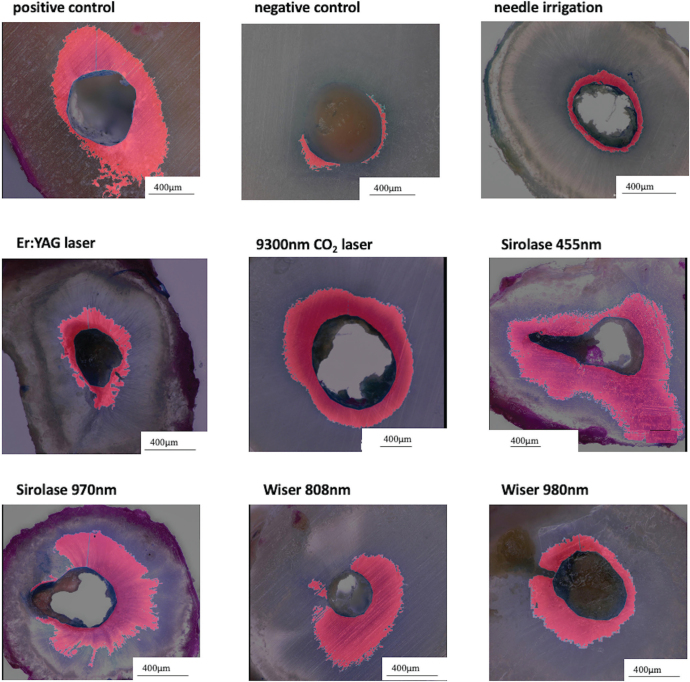
Digital microscopy photos of dye penetration in the apical root third in all experimental groups, ×200.

### Measurements of dye penetrated dentin area

The ANOVA test detected a statistically significant effect on the dye penetrated dentin area of both the activation method applied (*p* < 0.001) and the root region (*p* = 0.034). The interaction between the two factors was also statistically significant (*p* = 0.037).

The post-hoc analyses showed an overlapping ranking regarding the measurement of the penetrated dentin area for all root parts combined (Table 2): diode lasers (D455, D970, D808,) had the largest surface area of penetration, followed by Er:YAG (Er:YAG), positive control (POS) and CO_2_ laser (CO_2_). Less effect on penetration was observed in the negative control (NEG), diode Wiser 980 (D980) and conventional treatment (CONV) (Table 2 and Figure 5). Group D455 was hereby significantly different from group D980, ER:YAG and CO_2_ laser as well as from the control groups (POS, NEG, CONV). Conventional needle irrigation (CONV) penetrated the root dentin significantly lesser than Er:YAG, CO_2_, the positive control (POS) and diode lasers (D455, D970, D808, D980).

**Table 2:** Results of dye penetration (mean and standard deviation) based on Fisher’s LSD Test (*p* < 0.05). Different uppercase letters indicate statistically significant differences, with largest penetrated dentin areas depicted by letter A and decreasing up to H.

Group	Mean (SD)	Dye penetration area: ranking for entire root							
D455	2.3 (1.3)	A							
D970	2.1 (1.3)	A	B						
D808	1.9 (0.8)	A	B	C					
ER:YAG	1.67 (0,8)		B	C	D				
POS	1.55 (0.8)			C	D				
CO_2_	1.48 (0.75)			C	D				
NEG	1.21 (1.1)				D	E			
D980	1.16 (0.5)				D	E			
CONV	0.95 (0.4)					E			
		**Dye penetration area: ranking for the apical root third**							
D 455	2.90 (2.0)	A							
D 970	2.18 (1.8)	A	B	C					
D 808	2.07 (1.1)	A	B	C	D				
POS	1.61 (0.9)		B	C	D	E	F		
CO_2_	1.36 (0.8)			C	D	E	F	G	
Er:YAG	1.30 (0.5)			C	D	E	F	G	
D 980	1.09 (0.5)						F	G	H
CONV	0.52 (0.3)							G	H
NEG	0.34 (0.4)								H

SD: standard deviation

Regarding the coronal and middle root thirds, respectively, the values for the nine groups were not significantly different (Figure 5). In contrast, for the apical root part, significant differences were found between the groups with an overlapping ranking of diode lasers (D455, D970, D808) penetrating large dentin areas followed by the positive control (POS), Er:YAG, CO_2_ laser, conventional syringe irrigation (CONV), negative control (NEG) and diode laser Wiser 980 nm (D980) (Table 2 and Figures 5 and 6).

### Measurements of the maximal dye penetration depth

The ANOVA test detected statistically significant effects on the maximal dye penetration depth of the activation method applied (*p* < 0.001) and of the root regions (*p* = 0.029).

Measurements of the maximal penetration depth for all experimental groups decreased statistically significantly from coronal to middle and to the apical root thirds. Results of the post-hoc comparisons among the groups for the maximal dye penetration depth over the entire root length and the apical third can be seen in Table 3. Regarding the entire root length, diode lasers reached the highest values followed by the positive control (POS) and LAI with CO_2_ and Er:YAG laser. Traditional needle irrigation with EDTA and NaOCl (CONV) was similar to the negative control (NEG) and Er:YAG laser activation. The conventional method penetrated significantly less deep in the root dentin than POS, CO_2_ and diode lasers (Table 3).

**Table 3:** Results of maximal dye penetration area (mean, standard deviation) based on Fisher’s LSD Test (*p* < 0.05). Different uppercase letters indicate statistically significant differences, with largest penetrated dentin areas depicted by letter A and decreasing up to J.

Group	Mean (SD)	Dye penetration maximal depth: ranking for entire root									
D 455	599 (266)	A									
D 970	582 (347)	**A**									
D808	534 (150)	**A**	**B**								
D980	456 (246)		**B**	**C**							
POS	449 (148)		**B**	**C**							
CO_2_	432 (183)		**B**	**C**							
Er:YAG	378 (153)			**C**	**D**						
CONV	300 (180)				**D**						
NEG	291 (170)				**D**						
		**Dye penetration maximal depth: ranking for the apical root third**									
D 455	497 (318)	A	B	C	D	E					
D 808	440 (160)		**B**	**C**	**D**	**E**	**F**				
D 970	426 (253)		**B**	**C**	**D**	**E**	**F**	**G**			
POS	396 (170)			**C**	**D**	**E**	**F**	**G**			
D 980	316 (166)					**E**	**F**	**G**	**H**	**I**	
CO_2_	302 (140)						**F**	**G**	**H**	**I**	**J**
Er:YAG	242 (93)								**H**	**I**	**J**
NEG	146 (59)									**I**	**J**
CONV	121 (46)										**J**

SD=Standard deviation

Regarding the apical values separately, we noticed a significant difference between the conventional needle irrigation (CONV) and diode lasers as well as the positive control (POS) but not in comparison to Er:YAG and CO_2_ laser or the negative control (Table 3).

## Discussion

The aim of this study was to investigate the pure mechanical effect of LAI with a 9,300 nm CO_2_, and low-power diode lasers, on the penetration capacity of a dye into root dentin and to compare it to the effects of activation with an Er:YAG laser, conventional needle irrigation with EDTA and NaOCl as well as to control groups with maximal opened and occluded dentinal tubules.

The dye penetrated the dentin of all experimental groups to different degrees depending on the method applied but none of the applied methods reached full dentin wall depth.

Some authors question that irrigant penetration might not be only dependent on the smear layer and the irrigation protocol but also largely on the presence of tubular sclerosis in the dentin [21, 22]. Dentin sclerosis refers to a continuous decrease of the dentinal tubuli diameter with increasing age of patients. Studies showed that teeth of patients older than 60 years had an apical dentin penetration of less than 7% of the entire dentinal volume [21, 23–25]. The physiological phenomenon of sclerosis also seemed to have impacted the results of the experiments and thus present a limitation of this study. This can be derived from the incomplete and inhomogeneous dye penetration in the positive control group especially in the apical root third in that the root walls were etched with phosphoric acid in order to open a maximum of dentin tubule entrances. It is thus possible that applying the tested methods on teeth without dentin sclerosis might have shown significantly different results. An additional limiting factor of this study was the use of anonymous tooth samples. It was not possible to account for the differences of sclerosis due to age. However, it can be supposed that the tooth samples (wisdom teeth) were extracted from a pool of patients with a relatively small range of age.

Another important aspect to take into account is the physiological decrease in the number of dentinal tubules from 40.000 mm^−2^ in the coronal region to 14.400 mm^−2^ in the apical region [26, 27]. This might also explain the decrease in penetration of dyes from the coronal to apical region that could be seen in our results and the literature. The physiologically higher number of tubules with a greater diameter and comparatively large volume of irrigant/dye with high pressure in the main root canal generally led to deeper penetration coronally, and differences between the groups were less evident [28].

Although the smear layer did not seem to be a tight diffusion barrier for small molecules, the negative control was in all settings one of the groups with the poorest penetration. In particular, the negative control group showed less homogenic dye penetration along the root main canal compared to the other groups. In connection with the negative control, it is important to note that the smear layer might have not been homogeneously distributed all over the root dentin walls. This could be led back to the fact that mechanical preparation with round files is known to leave – dependent on the preparation diameter – important sizes of areas of the individual root diameter forms untouched [29].

Thus, it is not sure to which extent the results of dye penetration are finally impacted by the activation and cleaning with the different methods applied or by the simple absence of a smear layer in some areas.

Two additional samples served as controls for the dentin surface patterns. Their dentin micromorphology was examined under the SEM. This method did not allow for a longitudinal observation of the surface pattern as it would be possible with micro-CT imaging [30].

Using micro-CT might have provided for a better standardization of the preoperative volume of smear layer and could have contributed to a quantification of the smear layer which in turn might have explained the dye penetration of the underlying results.

Dye penetration in the positive control group showed a significantly larger surface of penetration compared to the negative control but was however inferior to experimental groups with diode lasers.

This might indicate that cleaning the dentin walls and dentinal tubule entrances from the smear layer with chemical and or mechanical measures can be an important aspect for dye propagation into deep dentin layers but additional aspects resulting from the pure mechanical effects of laser activation of water might be even more important.

The first null hypothesis stating that LAI with the tested diode and CO_2_ lasers would be at least as effective as or superior to traditional needle irrigation in dentinal tubule penetration could be accepted. Considering both, the dentin penetration area and the maximal depth of dye penetration, laser activation of ultrapure water was at least as effective as needle irrigation with chemicals such as NaOCl and EDTA.

In the apical root third, the most challenging region to clean, the dentin area of dye penetration after conventional needle irrigation with NaOCl and EDTA was similar to the dye penetration of the negative control. Moreover, the ranking for conventional needle irrigation also overlapped with the ranking of the results from laser activation of ultrapure water with Er:YAG and CO_2_ laser. Diode lasers and the positive control with maximal opened dentinal tubule entrances allowed for a significantly larger dye penetration. Regarding measurements of the maximal dentin penetration depth, the dye penetrated significantly deeper with the Er:YAG and CO_2_ laser as with conventional needle irrigation.

Conventional treatments with syringe irrigation face the problem of an apical vapor lock, affecting the irrigant from penetrating the apical region and reacting to the smear layer [31]. Penetration depth values of the underlying study go in line with values of the literature [11, 32].

Laser activation and resulting streaming in the irrigant basically pushes the vapor lock to travel coronally, enabling irrigant penetration in the apical region. However, it seemed that the mechanical effects of the streaming velocities and shear forces on the root walls resulting from laser activation with Er:YAG and CO_2_ lasers were not efficient in removing the smear layer and enhancing the dye to penetrate larger root dentin areas as in the conventional needle irrigation. This observation is also described in the literature, authors stated that the effects of Er:YAG laser activation of water do not lead to cleaning the root canal walls from smear layer [33]. Moreover, it was seen that the CO_2_ laser led to superficial melting of the root dentin and the smear layer that obstructed the dentinal tubules and might impact dye penetration [16].

Diode laser shown in the experiments significantly affected deeper and larger dentin areas with dye penetration compared to the other tested wavelengths. This is why the second null hypothesis stating that CO_2_ and diode laser activation would lead to effects similar to Er:YAG laser activation could only be accepted in part. CO_2_ and Er:YAG laser activation led to similar effects but diode lasers were significantly different.

The mechanism of diode laser activation is different from that of Er:YAG and CO_2_ lasers due to their different wavelengths and absorption capacities by transparent liquids. Er:YAG and CO_2_ lasers were activated within the pulpal chamber. Both lasers are very well-absorbed by water-based irrigants and introduce relatively large vapor bubbles and high streaming velocities [19]. Other investigations showed that the temperature increase of the irrigant after 20 s of activation within the coronal part of an artificial plastic root model ranged between 7°C with the Er:YAG and 15°C with the CO_2_ laser with 0.4 W power input [20].

Diode lasers, in contrast, were used as heaters with the modified black tips. The formation of ‘bubbles’ due to boiling of the liquid around the hot laser tip is a comparatively slow process, which was shown in the literature to lead to significant temperature increase of the liquid [20]. Diode lasers used as hot tips with a special black coating of the laser tip can introduce vapor bubbles in a water-based irrigant with low-power parameters [19]. The temperature increase measured in the coronal region could reach more than 25°C with 0.3 W power input [20]. The temperature in the apical regions might be even higher for diode lasers as the liquid volume around the laser tip decreases there. These findings suggest that the activation with diode lasers could heat the irrigant more than the application of other laser types.

Literature describes the fact that the viscosity of irrigants can be modulated by the liquid’s temperature. Increasing the temperature will decrease the viscosity and might, therefore, allow an easier and deeper penetration in tubules. This is why some authors suggest for clinical protocols to decrease the viscosity and the surface tension of irrigants, such as EDTA and NaOCl, in order to improve their penetration into the tissue and their chemical effects [20, 34].

The above-described results suggest that the same applied for the methylene blue dye. The temperature increase with diode lasers and the consecutive decrease of the viscosity of the dye might explain the present results and provide suggestions for potential improvements of disinfection with heated irrigants as reported in the literature [35, 36]. Differences between the diode lasers might be attributed to differences in the laser tip materials and adhesion of the black coating.

It is interesting to note that both the 9.3 µm CO_2_ and diode lasers were observed to melt the superficial dentin layer while activating water in a SEM study [16]. At first glance, this appears to conflict with the penetration results we observed. However, this phenomenon may be explained by the theory that the process of superficial dentin melting takes some time within which the irrigant is able to penetrate the tissue until the melted dentin obstructs the tubule entrances with recrystallized material.

Comparing the present results to those of other studies is challenging due to the wide variety of protocols and methodologies employed. Some studies evaluate the penetration of a dye [7, 11, 22], while others examine the penetration of the irrigant itself [10, 24, 37] both in horizontal [7, 11, 22, 24] and longitudinal sections [10, 37] of the root. Additionally, the use of various activation methods, laser wavelengths, power parameters and chemical differences between penetrating liquids must be taken into account, along with the age of the patients and the degree of dentinal sclerosis in the root samples. These parameters may lead to significantly different penetration patterns [23].

Absolute values of penetration should also be viewed with caution, as the color recognition system used for the calculation accounted for the presence of methylene blue only above a certain intensity of color, and regions with less intense coloration may have been missed even with dye present. Moreover, it is possible that the dye continued to penetrate the dentin after the group-specific rinsing protocol despite drying with paper points and storage of the samples in a vacuum desiccator. Despite this, the methodology is standardized and allows for comparison between the experimental groups.

In addition, we would like to clarify that endodontic irrigants such as NaOCl or EDTA may show dentin penetration patterns that are different from methylene blue. Endodontic irrigants and methylene blue dye have different physical properties such as viscosity and wettability that are directly linked to their penetration capacity. These parameters can further be influenced by the irrigant’s concentration and temperature [38]. On the basis of this experiment, it is thus not possible to conclude on the penetration of clinically applied irrigants which can be seen as a limitation of this study. However, we consider the results as important for future research that comes closer to the clinical situation and application.

The results may indicate a trend of increased penetration for LAI with diode lasers compared to traditional needle irrigation. The present results give hints that dyes activated with the tested irrigation methods do not reach deep dentin layers that might harbor bacteria and their by-products. Future research should test the combined effect of laser activation and chemical active agents such as NaOCl and EDTA on penetration and disinfection in root dentin.

## Conclusion

Based on the results of the present study, it can be concluded that it was not possible to achieve consistent penetration of the deep dentin layers that may harbor bacteria with methylene blue. Traditional needle irrigation using chelating agents allowed for a uniform penetration of the superficial dentin layers with methylene blue in the coronal and middle thirds, but less in the apical third of the roots. LAI facilitated deeper penetration of the dye into the root dentin, with the degree of penetration being dependent on the temperature generated by the laser. Diode laser demonstrated the highest levels of penetration especially in the apical root third. Er:YAG and CO_2_ laser activation without the application of NaOCl and EDTA were similar to traditional needle irrigation with NaOCl and EDTA in the apical root third.
